# Efficacy and feasibility of a digital speech therapy for post-stroke dysarthria: protocol for a randomized controlled trial

**DOI:** 10.3389/fneur.2024.1305297

**Published:** 2024-01-31

**Authors:** Yuyoung Kim, Minjung Kim, Jinwoo Kim, Tae-Jin Song

**Affiliations:** ^1^HCI Lab, Yonsei University, Seoul, Republic of Korea; ^2^HAII Corporation, Seoul, Republic of Korea; ^3^Department of Neurology, Seoul Hospital, Ewha Womans University College of Medicine, Seoul, Republic of Korea

**Keywords:** dysarthria, stroke, digital, speech therapy, application

## Abstract

**Background:**

Dysarthria is a motor speech disorder caused by various neurological diseases, particularly stroke. Individuals with post-stroke dysarthria experience impaired speech intelligibility, communication difficulties, and a reduced quality of life. However, studies on the treatment of post-stroke dysarthria are lacking. Digital speech therapy applications have the advantages of being personalized and easily accessible. However, evidence for their efficacy is not rigorous. Moreover, no studies have investigated both the acute to subacute, and chronic phases of stroke. This study aims to investigate the efficacy and feasibility of digital speech therapy applications in addressing these gaps in dysarthria treatment.

**Methods and design:**

This study is a multicenter, prospective, randomized, evaluator-blinded non-inferiority trial. We aim to recruit 76 participants with post-stroke dysarthria. Eligible participants will be stratified based on the onset period of stroke into acute to subacute, and chronic phases. Participants will be randomized in a 1:1 to receive either a personalized digital speech therapy application or conventional therapy with a workbook for 60 min daily, 5 days a week, for 4 weeks. The primary outcome is the improvement in speech intelligibility. This will be measured by how accurately independent listeners can transcribe passages read by the participants. Secondary outcomes, which include speech function, will be evaluated remotely by speech-language pathologists. This includes the maximum phonation time, oral diadochokinetic rate, and percentage of consonants correct. Participants’ psychological well-being will also be assessed using self-report questionnaires, such as depressive symptoms (Patient Health Questionnaire-9) and quality of life (Quality of Life in the Dysarthric Speaker scale). The trial will also assess the feasibility, participant adherence, and usability of the application. Rigorous data collection and monitoring will be implemented to ensure patient safety.

**Conclusion:**

This trial aims to investigate the efficacy and feasibility of digital speech therapy applications for treating post-stroke dysarthria. The results could establish foundational evidence for future clinical trials with larger sample sizes.

**Clinical trial registration:**

Clinicaltrials.gov, identifier: NCT05865106.

## 1 Introduction

Stroke is a leading cause of mortality and long-term disability (1). Stroke can elicit a range of neurological deficits or handicaps, including speech and language problems. Dysarthria is a neurologic motor speech disorder caused by disruptions in the cranial nerve and muscular control of the speech mechanism. It is estimated that approximately 22–60% of stroke patients experience dysarthria within the first week following a stroke (2–4). Approximately 35% of patients continue to exhibit dysarthria even after 6 months (5). Moreover, post-stroke dysarthria significantly impairs speech intelligibility and articulation movements (2). This impairment leads to substantial communication difficulties, emotional distress, social isolation, and poor quality of life (6, 7).

Interventions for dysarthria involve a range of strategies, such as neurorehabilitation, behavioral exercises, and social support (2). Behavioral speech exercises strengthen the breathing and oral muscles to improve speech control. Strategies such as slowing down speech or controlling pitch are also used to enhance speech intelligibility. The treatment plan should be personalized based on the patient’s prior communication ability, recovery stage, and needs (8).

Despite the critical need for effective treatment of dysarthria, traditional therapies are often limited by their monotonous repeated nature. Consequently, these challenges can result in reduced treatment adherence (9). Moreover, the significant time and effort required by physicians and speech-language pathologists (SLPs), often restricts patient access to necessary therapeutic resources (10). Studies have indicated that only approximately one-third of patients receive adequate speech therapy, with substantial variations in therapy frequency (11). Given these limitations, personalized dysarthria treatment becomes necessary because of the diverse patterns of speech impairment in post-stroke dysarthria caused by different stroke lesions (12). These observations highlight the urgent need for alternative approaches, such as personalized digital speech therapy.

Compared with traditional in-clinic methods, these digital applications offer substantial benefits. These include the ability for patients to engage in various speech exercises from home. They improve treatment precision, enhance economic efficiency, ensure patient safety, maintain continuity of care, and support self-management (13, 14). This approach reduces the need for clinic visits and related expenses (15, 16). Furthermore, digital therapy is especially beneficial during the COVID-19 pandemic or endemic as it allows patients to continue their therapy without interruption (17).

Additionally, there is a noticeable lack of high-quality, comprehensive research on the treatment of post-stroke dysarthria. Many existing randomized controlled trials on post-stroke dysarthria have been on a small scale, limiting their ability to provide conclusive evidence regarding the effectiveness of various treatments (18). This significant research gap highlights the importance of our study, which aims to investigate both the efficacy and feasibility of a personalized digital speech therapy application for participants with post-stroke dysarthria.

This study aims to investigate the efficacy and feasibility of a digital speech therapy application for patients with post-stroke dysarthria. Ultimately, our trial seeks to establish our digital speech therapy application as a comprehensive solution. Our application strives to enhance the accessibility and practicality of post-stroke dysarthria. It provides personalized treatments tailored to each patient’s unique needs, thereby overcoming the limitations of traditional rehabilitation methods (12). We hypothesize that our digital speech therapy application would be non-inferior to conventional speech therapies in enhancing patient speech intelligibility and function for post-stroke dysarthria. Additionally, we anticipate positive impacts of our digital speech therapy application on psychological well-being, such as reduced depression and improved quality of life in patients with post-stroke dysarthria. Furthermore, this trial will assess the feasibility of this innovative approach in a clinical setting. It will focus on critical aspects such as recruitment and retention rates, participant adherence, safety, and gathering user feedback.

## 2 Methods and analysis

### 2.1 Trial design

**Figure 1:**
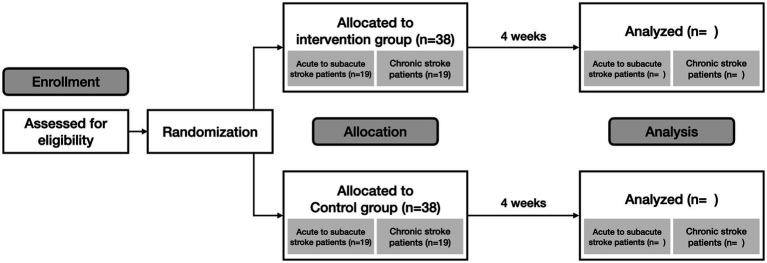
Study design.

This study is a multicenter, prospective, randomized, evaluator-blinded, non-inferiority trial designed to evaluate the efficacy and feasibility of a digital speech therapy application for post-stroke dysarthria (Figure 1). Participants will be recruited from three stroke centers in South Korea: Ewha Womans University Seoul Hospital, Mokdong Hospital, and National Rehabilitation Center. The trial will be conducted in accordance with the Declaration of Helsinki (19) and received ethics approval from the Institutional Review Board (Approval number: EUMC 2023–02-002, and NRC-2023-01-007). It is registered on clinicaltrials.gov as NCT05865106.

Participants will be recruited based on eligibility criteria (Table 1). Prior to enrollment, participants will receive a detailed explanation of the trial. Following this, written informed consent will be obtained from all participants. Subsequently, the participants’ demographic information and medical histories will be collected. Next, the participants will be randomly assigned to either the intervention or the control group. Randomization will be stratified based on the acute to subacute, and chronic phases of stroke.

**Table 1:** Inclusion and exclusion criteria.

Inclusion criteria	Justification for inclusion criteria
Aged 18 or over	To maximize the recruitment pool, our age inclusion criterion is set for individuals over 18 years old. However, given the nature of stroke and its higher prevalence in older populations, the majority of our participants are expected to be likely 50 years old or older.
Neurologically stable stroke patients diagnosed by a stroke specialist neurologist and demonstrate no changes in their total National Institute of Health Stroke Scale over 72 h.	Neurologically stable stroke patients suitable for clinical trials can be determined by a clinician to ensure reliable and safe participation during clinical trial.
First-ever stroke patients without previous stroke history.	The efficacy of a digital speech therapy application is hard to confirm for dysarthria caused by pre-existing stroke sequelae.
Paticipants with sufficient cognitive abilities to operate the digital speech therapy application [Korean Mini-Mental State Exam score ≥ 26 (20)].	Cognitive ability can directly influence the patient’s speech improvements and confound results.
As judged by the stroke specialists: participants with sufficient vision [visual acuity and visual field test (21)], hearing [Weber test and Rinne test (22)], communication skills, and motor skills to participate in this study.	Stroke specialists will evaluate potential participants for sufficient vision, hearing, communication, and motor skills. This evaluation will ensure that participants have the necessary abilities to use the digital speech therapy application without difficulty.

Exclusion criteria	Justification for exclusion criteria
Co-existing language disorder (e.g., aphasia). Aphasia will be determined by a stroke specialist using the National Institute of Health Stroke Scale (including fluency, comprehension, repetition, and naming test).	Co-existing language disorders could hinder study participation and cause confounding results.
Co-existing progressive neurological disorders that can affect dysarthria (e.g., dementia, Pick’s disease, Huntington’s disease, Parkinson’s disease, or Parkinsonism).	A major medical comorbidity, including cognitive dysfunction, could interfere with appropriate and reliable performance of intervention or cause confounding results.
Diagnosis of severe mental disorders as determined by a clinician (e.g., schizophrenia, alcohol addiction, or drug addiction).	In order to reliably utilize the application, it is necessary to exclude those with severe mental illness, as determined by the clinician. Mental disorders will be assessed according to participants’ history and current taking medications such as antipsychotics.
Participants taking concomitant medications that could affect the trial results during the study period (e.g., cognitive dysfunction medications, anticholinergics, anti-epileptic drugs, anti-anxiety drugs, antidepressants, antipsychotics, and hypnotics).	Medications that can affect cognitive or emotional function could cause confounding results. However, it is permitted if continued use has been at a stable dose for 2 months prior to randomization in the case of anti-anxiety drugs, antidepressants, and hypnotics.
Participants unable to use/access smartphone technology according to our digital speech therapy application protocol.	The intervention will be provided through the participant’s smartphone application.
Illiterate patients.	Participants must be able to read and understand the intervention delivered by the application.
Participants unable to communicate in Korean.	The application will only be available in Korean language.

### 2.2 Inclusion procedure

The principal investigator will screen and exclude participants with abnormalities in oral structures based on speech assessments. A comprehensive list of eligibility criteria is presented in Table 1. Stroke specialists will identify participants with dysarthria using the National Institute of Health Stroke Scale (NIHSS) criteria (23). The NIHSS is utilized for its ease of administration and standardized approach, which minimizes interpersonal variation in assessments. Also, the sub-section of “10. speech” is suitable for initially identifying dysarthria severity in stroke patients. We aim to recruit participants with both mild-to-moderate and severe dysarthria. Mild-to-moderate dysarthria is characterized by slurred speech that remains understandable, and severe dysarthria involves mostly unintelligible speech or the inability to speak without evidence of aphasia. The eligible participants will be referred to a research coordinator to verify their suitability for the trial. To educate the participants, the principal investigator will provide detailed information about the study, such as intervention options, associated risks, and expected benefits. All recruitment and screening processes will be thoroughly documented.

Participants will be clearly informed that they have the right to withdraw from the study at any point without any consequences to their future medical care. Withdrawal criteria will include voluntary withdrawal by the participant, a usage rate falling below 40%, a need for immediate medical care that precludes continued participation, and any adverse events that are directly related to the digital therapy application. Additionally, the research team reserves the right to withdraw participants if there is significant noncompliance with the study protocol or if their health status changes to the extent that continued use of the application is deemed unsuitable or unsafe.

### 2.3 Randomization

Participants will be randomly allocated in a 1:1 ratio to either the intervention group or control group. An independent third-party entity will oversee the randomization process to ensure impartiality.

Additionally, the trial will stratify participants based on the onset period of their stroke: acute to subacute phase (within 1 month of onset) and chronic phase (from 1 month to 6 months of onset) (24). After confirming participants’ eligibility, we will assign each participant a sequential number for group allocation. For each stratum, we will actively allocate participants in a 1:1 ratio to either the intervention or the control group. A secure computerized system will generate an unpredictable and concealed allocation sequence. A blinded researcher will manage this sequence in opaque envelopes, which will be opened after the participant’s enrollment and baseline measurements are complete.

To ensure group balance and methodological rigor within each stratum, we will use permuted blocks of sizes two and four (25). The block size and allocation sequence details will remain confidential and inaccessible to the team members involved in recruitment, treatment, and assessment until the conclusion of the study.

### 2.4 Blinding

Due to the nature of speech therapy, the complete blinding of participants to their assigned interventions is challenging. We will implement evaluator blinding to mitigate this issue and ensure unbiased outcome assessments (26). Evaluators will be thoroughly trained to understand the study protocol and recognize potential biases. Their role is to analyze the trial results objectively without influence from other factors. Evaluators will not interact face-to-face with participants. Instead, they will remotely listen to patient recordings via a web system and evaluate the outcomes. This method ensures evaluators focus exclusively on outcome assessment. They will remain unaware of the participants’ group assignments and will not be involved in other stages of the research.

### 2.5 Intervention methods

#### 2.5.1 Intervention group

Participants in the intervention group will use a digital speech therapy application designed explicitly for post-stroke dysarthria. Participants will use the application independently, without requiring continuous assistance from caregivers or medical professionals. In addition, occasional guidance or support from family members will be permitted. As part of our regular monitoring process, researchers will follow up on the extent of family assistance to ensure it remains appropriate and consistent for the therapy protocol. However, the primary aim is for participants to manage the therapy autonomously. This approach promotes independence and self-reliance during treatment. Participants will engage in speech therapy for 60 min each day, 5 days a week, for 4 weeks. The flexibility of the application should enable participants to complete their therapy in either a single session or multiple sessions throughout the day. Before therapy begins, researchers will introduce the application to participants and guide them through its usage. Additionally, we will provide a manual booklet to ensure that participants can use the application confidently and independently.

As the majority of stroke patients are older adults, the application is designed to be user-friendly (27). It features elder-friendly functionalities and provides progress updates through both text and voice descriptions to ensure accessibility. Design elements such as button size and spacing are tailored to accommodate the potential fine motor and vision challenges faced by older users (28).

Given the variability in speech impairment patterns due to different stroke lesions in post-stroke dysarthria (29), this application provides a personalized approach to speech therapy. When participants log into the application, it prompts them to perform four speech assessment tasks to evaluate their current speech conditions. The application uses a smartphone microphone to measure the ambient noise levels before each task to ensure optimal recording conditions. The assessment proceeds only if the noise level is <50 dB. Four assessment tasks were adapted from traditional dysarthria assessments. Each task comes with detailed instructions and sample demonstrations to help participants perform assessments efficiently.

- **Sustained Vowel:** Participants are instructed to sustain the vowels/a/, /i/, and/u/, using their regular speaking tones. Participants are to perform the task twice, and the longer attempt between the two is recorded for analysis (30).
- **Diadochokinetic (DDK) Rate:** Participants are required to rapidly repeat syllables /pǝ/, /tǝ/, and /kǝ/, along with the sequence/pǝtǝkǝ/ each for at least 5 s. Subsequently, their performance is recorded for analysis (31).
- **Word Reading:** Participants are asked to read a set of 30 words from the Urimal Test of Articulation and Phonology 2 (UTAP2). The UTAP2 is specifically designed to assess articulation accuracy in Koreans (32).
- **Passage Reading:** Participants are instructed to read the passage in a comfortable voice at a natural pace. This assessment evaluates the patient’s articulation and overall speech intelligibility. The *Gaeul* Passage is the standard for examining motor speech disorders in South Korea (33).

**Figure 2:**
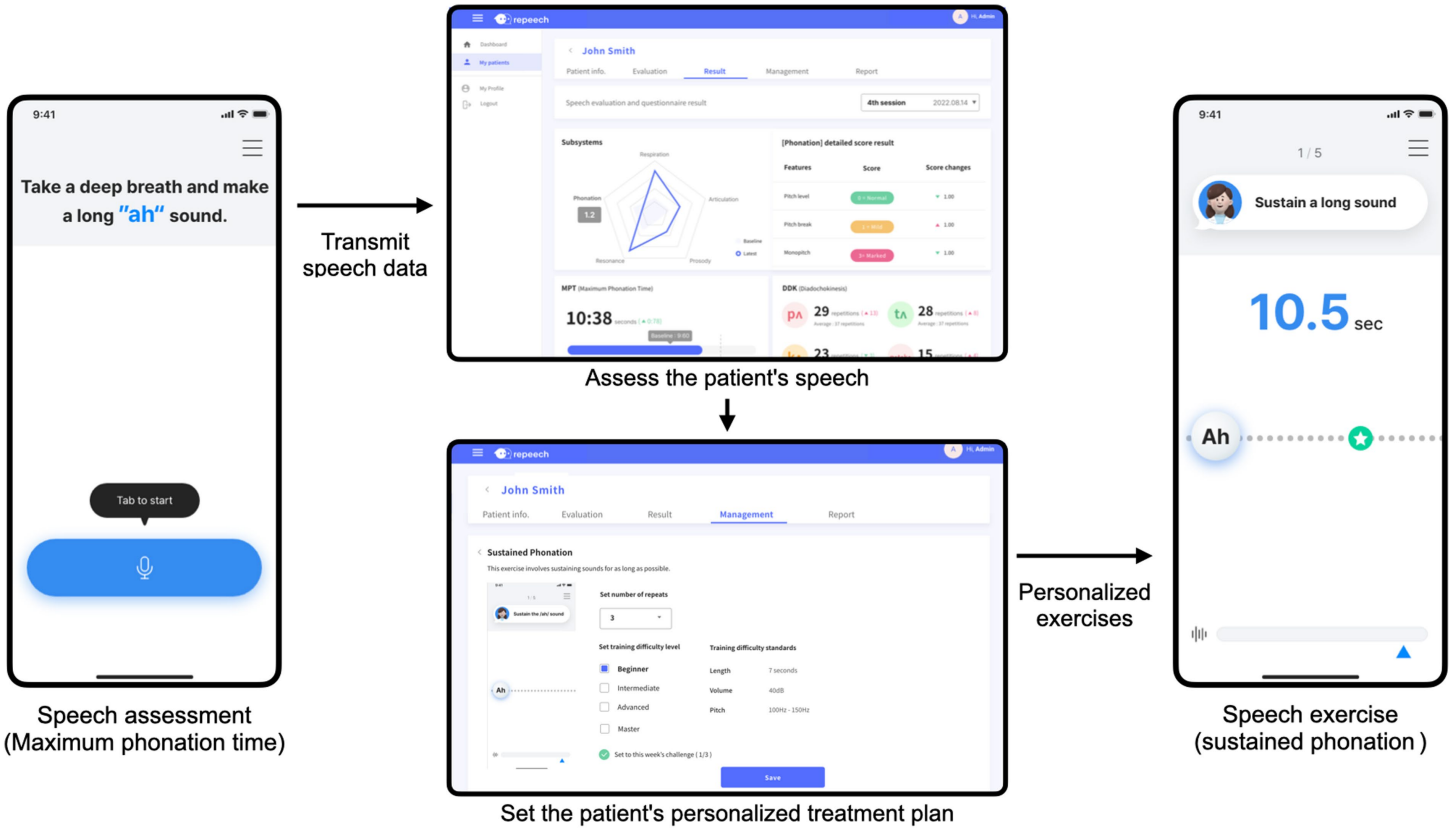
Digital speech therapy application system overview.

The recorded speech assessment data will be sent to a web system. The SLPs will review the recorded assessments remotely. This process enables them to identify specific patterns of dysarthric speech impairment using a web system, as shown in Figure 2. Based on these evaluations, a personalized treatment plan specifies the types and levels of exercise required. As an illustration, participants with respiratory impairment may be assigned the “sustained phonation” exercise. SLPs can also set target goals, including phonation duration, volume, and repetition. Conversely, participants struggling with articulation may perform reading exercises. Considering the common articulation error patterns observed in participants with dysarthria, we arranged the minimal pair sets. If a patient has difficulty stopping sounds, the SLP can set minimal pairs such as/p/ and /f/, or/b/and/v/. This plan is then uploaded to the patient’s application. Subsequently, the patient performs the prescribed speech exercises. The therapeutic goals are set at the beginning of each week. Based on the patient’s performance, the difficulty level of the exercises is adjusted during weekly reviews. If a patient completes the therapy tasks for 1 week, the subsequent week’s exercises are prescribed at a higher difficulty level. This adjustment ensures the continuous progression and adaptation of the patient’s improved speech skills.

Our digital speech therapy application integrates a comprehensive behavioral speech therapy exercise based on the established literature (2, 34). An introductory video at the beginning of the exercise explains the participant’s objectives and processes. The application provides real-time visual and auditory feedback during exercise to enhance participants’ immediate responsiveness. Moreover, the application utilizes a sophisticated signal processing algorithm to analyze voice data captured via the smartphone’s microphone. This algorithm assesses various speech acoustic variables, such as sound volume, pitch, reading speed, and pronunciation accuracy. Each exercise comes with predefined thresholds against which participant performance is measured. This enables the application to offer immediate and personalized feedback based on the individual’s progress and performance against these benchmarks. Additionally, the participants can listen to their recordings and receive additional feedback. This comprehensive feedback mechanism promotes patient engagement and constant improvement in speech therapy (35). Detailed procedures for the speech exercises are provided in Supplementary material.

Maintaining adherence and continuing to participate in the study is a crucial goal. Researchers will systematically offer direct feedback to participants regarding their performance and adherence. This approach encourages continuous patient engagement throughout the therapeutic process. To maintain patient adherence, the researchers will monitor the daily progress of each patient. Researchers will contact participants whenever a decrease in the usage of the application is observed. They will reach out through text messages or phone calls to understand the reasons behind the reduction in their usage. The researchers will then provide encouragement and support to reengage participants in the therapy process. Additionally, the application features daily and monthly calendars for visual progress tracking. It also records the duration of daily activities, fostering motivation and consistency among the participants. Moreover, the participants are encouraged to contact the research team for assistance with application usage challenges, including user errors and interface navigation. Researchers will promptly respond to concerns communicated via phone, email, or text to offer immediate support in resolving issues.

#### 2.5.2 Control group

Participants allocated to the control group will undergo conventional therapy for post-stroke dysarthria over 4 weeks. In the absence of a standard protocol for post-stroke dysarthria treatment in South Korea, we have developed a conventional speech therapy workbook. This workbook integrates various clinically validated behavioral therapy techniques (2, 34). Its content is aligned with the intervention group’s digital speech therapy application to ensure consistency in treatment approaches. Participants will engage in the workbook for 60 min daily, 5 days a week, for 4 weeks to match the intervention group’s treatment dose and frequency. The workbook ensures treatment consistency across the multiple centers involved in this clinical trial. Before starting therapy, the researchers will guide the participants using the conventional therapy workbook.

In line with the intervention group, occasional guidance or support from family members will be allowed. This approach ensures that both groups have access to similar levels of support. By doing so, we promote equality in the treatment conditions across the groups. During regular follow-ups, researchers will monitor the extent and nature of any assistance family members provide. This monitoring will help ensure that the level of support remains balanced and appropriate for both groups.

Researchers will conduct daily follow ups to ensure adherence to conventional therapy workbook. During these interactions, researchers will discuss the participants’ progress, address challenges, and provide support and encouragement to maintain consistent participation in therapy. This approach ensures that the control group receives the same level of attention and support as the intervention group, thereby maintaining trial integrity. After the clinical trial, participants in the control group will also have access to the digital speech therapy application.

### 2.6 Outcome measurements

The study will span 4 weeks, and the details are provided in Table 2. Three assessment stages will be conducted during the process.

**Table 2:** Schedule of enrollment, interventions, and assessments.

	Enrollment	Randomization and allocation	Post-allocation		Close-out
Timepoint	Pre-baseline		Baseline	4 weeks	Post-study
Enrollment					
Eligibility screen	X				
Informed consent	X				
Allocation		X			
Interventions					
Conventional speech therapy			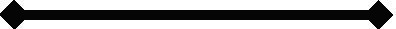		
Digital speech therapy application			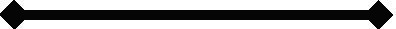		
Assessment					
Speech intelligibility			X	X	
Percentage of consonants correct			X	X	
Oral diadochokinetic			X	X	
Maximum phonation time			X	X	
Depression			X	X	
Quality of life			X	X	
System usability					X

Demographic and clinical characteristics will be recorded after enrollment in the study. This initial recording includes demographic data (age and sex), medical history, stroke information (type and location of stroke and post-stroke duration), level of impairment measured using the NIHSS (23), cognitive status determined using the Korean Mini-Mental State Examination (K-MMSE) score (20), and Clinical Dementia Rating (CDR) tests (36). The demographic and clinical characteristics of the participants are shown in Table 3.

**Table 3:** Variables evaluated at baseline and study period.

Components	Subcategories
Age	Years
Gender	Male / Female
Medical history	Medication history, concomitant disease history (for example, dementia [Alzheimer’s disease, vascular dementia], central nervous system infection [e.g., human immunodeficiency virus, syphilis, Creutzfeldt-Jakob disease], Pick’s disease, Huntington’s disease, Parkinson’s disease, and others).
Medication	Details of concomitantly administered drugs (for example, medications for dementia treatment, central nervous system stimulants, anticholinergics, tricyclic antidepressants, typical antipsychotics, sleeping pills, typical antipsychotics, anxiolytics, antidepressants, and thyroid hormone)
Type of stroke	Ischemic/hemorrhagic
Stroke lesion location	Cortical (for example, frontal, parietal, and so on), subcortical, cerebellum, thalamus, basal ganglia, and so on.
Side of stroke involvement	Left/Right
Time to enroll after stroke	In days
Cognitive function	Korean Mini-Mental State Exam, Clinical Dementia Rating
Neurological status	National Institute of Health Stroke Scale
Visual function	Intact/Non-intact
Hearing function	Intact/Non-intact
Aphasia	Yes/No

Subsequently, the participants will undergo a comprehensive assessment for post-stroke dysarthria. Psychological well-being questionnaires will also be administered to assess depression and quality of life. Participants will engage in the post-evaluation phase 4 weeks after the baseline assessment. We will repeat the speech assessments and psychological questionnaires from the baseline evaluations. Additional evaluations of usability and adherence rates will be conducted.

#### 2.6.1 Outcomes

The primary outcome is the change in speech intelligibility following the intervention. Speech intelligibility is crucial for effective communication and reflects how well a listener understands a patient’s speech (37). Speech intelligibility will be calculated using a passage-reading assessment. Participants will be instructed to read a passage aloud comfortably and naturally using the application for assessment. The application will record their speech and automatically upload the recordings to a secure server. Next, three independent SLPs will listen to the recordings via a web system. After hearing the passage once, they will transcribe it as they understand it. We will compare these transcriptions with the original passages to calculate the percentage of correctly transcribed words. Each participant’s final score will be the average of the scores of all three evaluators (38).

For secondary outcomes, the study will assess the participants’ speech function and psychological well-being through voice recordings and surveys via the application. The SLPs will listen to the recorded voices of the participants and rate each speech function measure. The maximum phonation time (MPT) assesses the ability to sustain vowel sound (39). Oral diadochokinetic (DDK) rate evaluates the speed, regularity, and accuracy of articulatory movements, with performance quantified by the number of syllables repeated rapidly and accurately (31). The percentage of consonants correct (PCC) is determined by comparing transcriptions from word-reading tasks with the original text to objectively assess the accuracy of consonant sounds (32, 40).

Self-report questionnaires will be used to assess psychological well-being. The Patient Health Questionnaire-9 (PHQ-9) evaluates the severity of depressive symptoms (41). The Quality of Life in the Dysarthric Speaker (QOL-Dys) scale quantifies how dysarthria affects various aspects of life. It specifically assesses the impact on daily activities (42, 43). These measures provided vital insights into the effects of interventions on communication and psychological health.

#### 2.6.2 Feasibility

A comprehensive evaluation will be conducted to assess the feasibility of this trial. First, the trial will estimate the number of potential participants, the duration of recruitment, and the success rate of screening to secure a representative sample size. Additionally, retention rates will be monitored throughout the trial. These measurements will ensure a rigorous sample size for future studies. Second, the participants’ adherence to digital therapy applications will be measured. This will include tracking the frequency and completeness of the application usage and participant engagement with the treatment protocol using a robust data collection and management system. Third, participant feedback will be collected to assess the acceptability and effectiveness of the digital therapy. We will use the System Usability Scale (SUS) questionnaire to measure the usability of the digital speech therapy application in terms of efficiency and user satisfaction (44). Additionally, interviews will be conducted with the application user to collect qualitative insights such as participants’ experiences, challenges, and suggestions for improvement. This comprehensive feedback will aid in a better understanding of the application’s usability and effectiveness and contribute to future development and enhancements. Finally, we will monitor patient safety and potential adverse events and uphold ethical standards. Digital speech therapy applications include the real-time monitoring of patient log data to swiftly identify and address usage errors or technical issues. Rigorous testing of the application’s performance will also focus on stability and user interface to assess its impact on therapy effectiveness and user experience. This approach ensures the safety and reliability of the digital tools throughout the study.

### 2.7 Analysis

The primary objective of this trial is to investigate the non-inferiority of the intervention group compared to the control group in terms of changes in speech intelligibility from baseline to post-treatment. The hypothesis is as follows:


$$H_{0}:\mu_{t}-\mu_{c}<\delta \;vs\;H_{1}:\mu_{t}-\mu_{c}\geq \delta$$

Where *μt* is the mean change in speech intelligibility score in the intervention group using the digital speech therapy application, and *μc* is the mean change in the control group receiving conventional speech therapy. It is assumed that there is no difference between the two groups, and this study seeks to establish the non-inferiority of the groups. The non-inferiority margin *δ* is set at 19 points, based on the assumption that a difference of 1 to 19 points on the speech intelligibility scale is clinically non-inferior (45).

The sample size required for the non-inferiority trial was determined using a statistical formula (46). This involved setting the significance level (alpha) to 0.025 for a one-sided test and targeting a power of 80% (*β* = 0.2) (47) with a standard deviation of 24.9 (48). This calculation revealed that 28 participants are required for each group. A total of 56 participants are required for the trial. To account for a possible dropout rate of 30%, we aim to recruit a total of 76 participants, with 38 participants in each group.


$$n=\frac{2{\left(z_{\alpha }+z_{\beta }\right)}^{2}\sigma^{2}}{{\left(\mu_{t}-\mu_{c}-\delta \right)}^{2}}\approx 28$$

Analyses will be performed using SPSS software (version 27; IBM Corp., Armonk, New York). Descriptive statistics will summarize the demographic data and outcome measures. For interval estimates, 95% confidence intervals will be provided. An intention-to-treat (ITT) analysis will include all randomized participants. Additionally, we will conduct a per-protocol (PP) analysis which includes only participants who fully adhered to the intended protocol.

First, the Shapiro–Wilk test will assess of the normality of the distributions. Based on these results, the appropriate parametric (independent two-sample t-test) or non-parametric test (Mann–Whitney U test) will be chosen for between-group comparisons. Then, mixed repeated-measures analysis of variance (ANOVA) will evaluate the changes over time within subjects and between groups, with fixed effects for time, group, and their interactions. Mauchly’s test will check sphericity by applying the Greenhouse–Geisser, if necessary. For significant findings, Tukey’s HSD tests will conduct post-hoc analyses with Bonferroni correction to control for the overall type I error rate at 0.05. The benchmark for statistical significance is set at *p* < 0.05.

We will address missing data using multiple imputation techniques and the Last Observation Carried Forward (LOCF) method. This approach aims to minimize bias and ensure the robustness of efficacy outcomes (49). Subgroup and sensitivity analyses will be conducted to explore the effects of participant characteristics and institutional factors on the primary efficacy results. In addition to the primary analysis, we will conduct subgroup analyses to examine the efficacy of our digital speech therapy application in various settings. Subgroup analyses include comparing its use during hospitalization with post-discharge home use. These analyses aim to provide a deeper understanding of how the setting influences the effectiveness of the therapy.

Furthermore, additional subgroup analyses will focus on the effects of the interventions across different stroke phases. Since participants are stratified based on the onset period of their stroke into acute to subacute, and chronic phases, these subgroup analyses will explore how the efficacy of the intervention varies between these two distinct stroke phases. We will perform regression analyses within each group stratified by the stroke onset period. If the statistical analysis reveals significant mean differences between various groups, post-hoc tests will be conducted to identify the specific groups among which these differences exist. This approach will provide valuable insights into whether the timing of a post-stroke intervention influences the efficacy of digital speech therapy. No interim analyses are planned to maintain the integrity of the non-inferiority margin or control for type I error rate.

## 3 Discussion

This protocol aims to evaluate a digital speech therapy application for post-stroke dysarthria in a randomized clinical trial. This will establish the efficacy and feasibility of the intervention and contribute vital data to inform future trials. The scarcity of rigorous research on post-stroke dysarthria treatment means that these findings will be significant for researchers and clinicians in this field (18, 50).

Previous research has demonstrated the efficacy of behavioral speech treatment in patients with chronic post-stroke dysarthria. For instance, previous study showed a significant improvement in patients with post-stroke dysarthria after 16 sessions over 4 weeks (51). Similarly, another study reported positive outcomes from 60-min sessions administered four times a week for 1 month in patients with at least 6 months of post-stroke dysarthria (52). However, these studies may not fully address error patterns and stroke lesion characteristics across stroke recovery phases (53–55). They recognized the importance of early intervention, which has been shown to significantly enhance outcomes (34, 56, 57). Our protocol aims to fill this research gap by stratifying patients into acute to subacute, and chronic phases. This approach offers a comprehensive view of the effect of treatment on the stroke recovery spectrum.

Our study proposes digital speech therapy designed to address the limitations of traditional in-clinic or in-hospital treatments for post-stroke dysarthria. Considering the diverse error patterns in participants with post-stroke dysarthria, our system emphasizes the delivery of personalized therapies through speech assessment. We aim to enhance treatment efficacy and improve patient adherence using a patient’s smartphone application.

Maintaining long-term commitment to post-stroke treatment can be challenging (58, 59). Patients may exercise less frequently than suggested (60), struggle to achieve daily treatment goals (61), or discontinue therapy entirely (59). Digital solutions offered for comfort in patients’ homes are expected to encourage more intensive speech treatment (6) and better adherence. Digital speech therapy applications enable easier access to speech therapy, increase engagement, and may reduce costs (62, 63). However, this study has several limitations. First, the limited sample size might limit the applicability of our results. Second, our digital speech therapy application is designed exclusively for Koreans and targeted at native Korean speakers. This language limitation could limit the generalizability to non-Korean populations. Third, the lack of direct supervision by clinicians or SLPs in digital speech therapy interventions could affect patient motivation and result in higher dropout rates (59, 64). Finally, a significant challenge arises from the high proportion of older participants, which is typical in stroke patient demographics (65). Despite considering the design of our application for the elderly population, these participants may experience lower usability and adherence to digital treatment because of their unfamiliarity with digital technology.

Despite these limitations, this study has several strengths. First, it aims to provide new evidence for the use of digital speech therapy in the treatment of post-stroke dysarthria. This could increase the adoption of digital treatment in the field. Second, the study is designed to investigate whether a digital speech therapy application is effective and feasible for stroke participants, regardless of their severity and recovery stage.

In conclusion, this trial explores the efficacy and feasibility of digital speech therapy applications in treating post-stroke dysarthria. Our study aims to provide significant insights that will aid future research by refining effect size estimates and enhancing power analysis. By addressing the complexities of post-stroke speech therapy through a digital application, we expect to enhance treatment strategies. Through our trial, we expect to be able to provide evidence of digital therapeutics for the treatment of post-stroke dysarthria.

## Ethics statement

The studies involving humans were approved by the EWHA Womans University Seoul Hospital Institutional Review Board (EUSMC-2021-12-011) and the National Rehabilitation Center Institutional Review Board (NRC-2023-01-007). The studies were conducted in accordance with the local legislation and institutional requirements. The participants provided their written informed consent to participate in this study. Written informed consent was obtained from the individuals for the publication of any potentially identifiable images or data included in this article.

## Author contributions

YK: Conceptualization, Methodology, Software, Writing – original draft, Writing – review & editing. MK: Writing – original draft, Writing – review & editing. JK: Funding acquisition, Writing – review & editing. T-JS: Supervision, Writing – review & editing.
