# Supplementary material for: Efficacy and feasibility of a digital speech therapy for post-stroke dysarthria: protocol for a randomized controlled trial
Source: Front Neurol. 2024 Jan 31;15:1305297. doi: 10.3389/fneur.2024.1305297 (PMC10865504; doi:10.3389/fneur.2024.1305297)
Supplement: Supplementary file 1 [file Data_Sheet_1.docx]

Supplementary Material

# Overview of speech therapy exercises

The application offers personalized speech therapy exercises based on speech evaluation results. These evaluations are performed through four speech tasks to analyze respiration, phonation, prosody, articulation, and resonance.

Each patient may have a different lesion and speech impairment error pattern. A skilled speech language pathologist listens to the patient’s recorded voice of the speech tasks and evaluates it. And then prescribes customized treatment according to the speech disorder pattern. For example, the speech language pathologist may determine the treatment priority based on the degree of decline in the patient’s five speech subsystems (respiration, phonation, resonance, articulation, and prosody) through a web (data logging) system and establish the difficulty and dose of each treatment. Consequently, the patient will undergo speech exercises tailored to their specific needs.

| **Speech exercises** | **Procedure** |
| --- | --- |
| 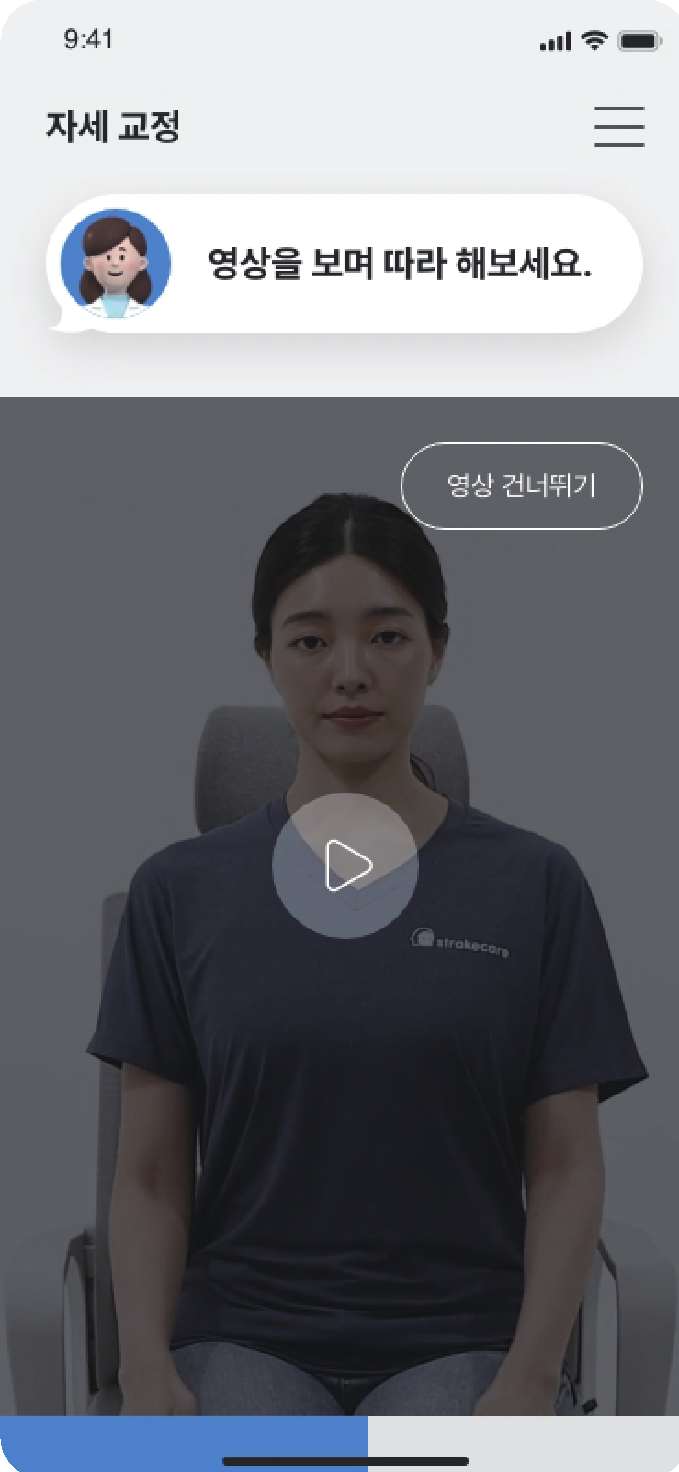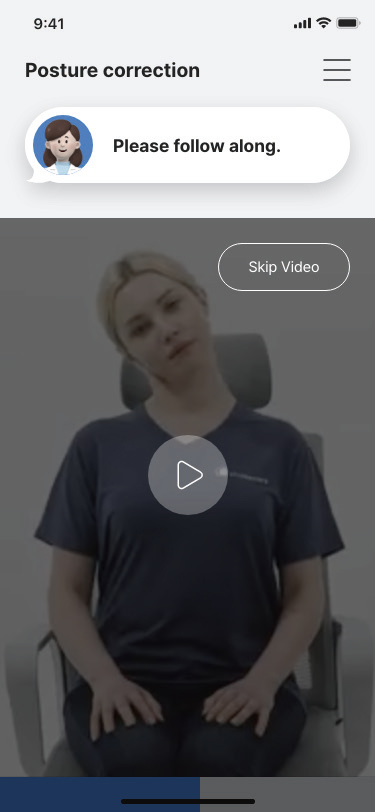 | **1. Posture correction**  Before starting speech exercises, the application will provide an instructional video to help patients make postural adjustments. The patient should follow the instructions to achieve the correct posture, such as sitting upright, to improve breath support for speech. |
| 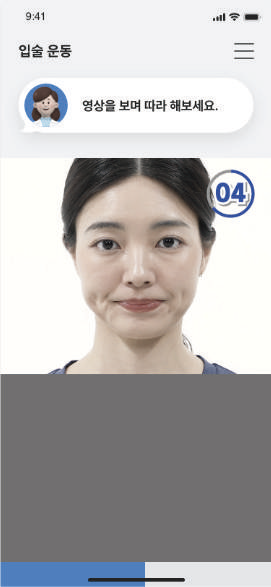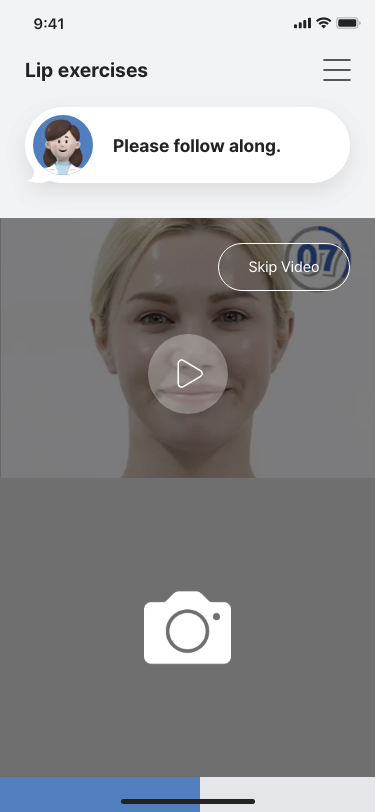 | **2. Oral motor exercises**  This exercise provides instructional videos for lip, cheek, jaw, and tongue exercises. The patient should be able to pause, skip, and play each video. Once the patient watches all the videos, he/she can watch the selected videos again. |
| 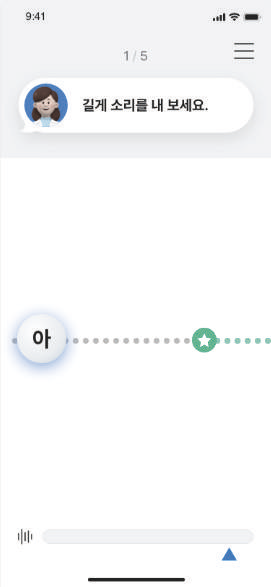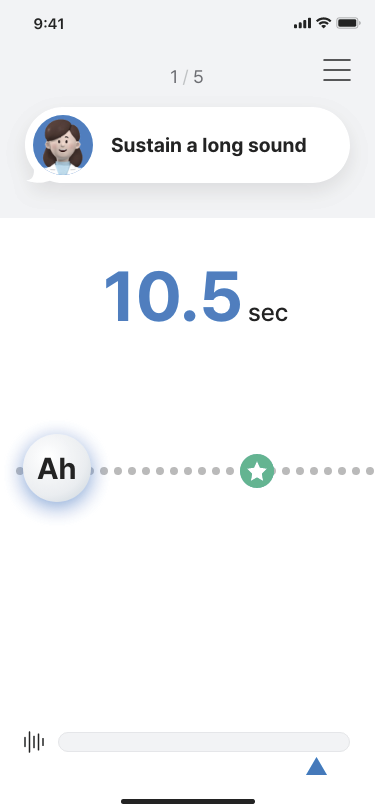 | **3. Sustained phonation**  This exercise requires the patient to loudly sustain the target sound (e.g., /ah/) for a long time. During the training, real-time feedback of sustained time (in seconds) is provided. The agent provides visual feedback if the patient exceeds their previous record. Once the patient completes the exercise, the result is provided, and they can listen to their recorded voice and decide to repeat the exercise. |
| 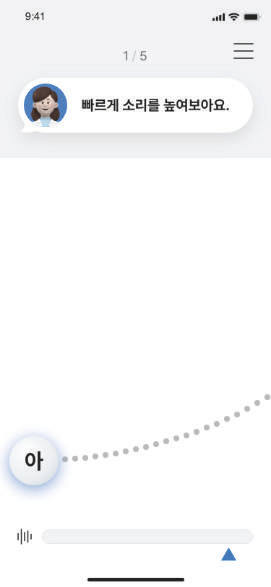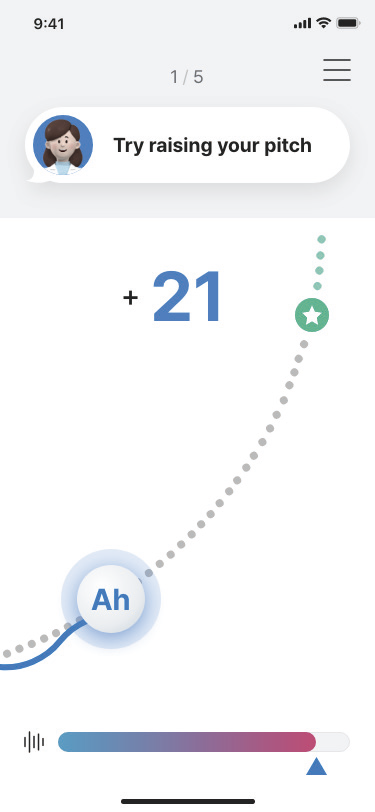 | **4. Gliding up**  This exercise requires the patient to quickly glide up the target sound (e.g., /ah/). During the training, real-time feedback of frequency (Hz) is provided. The agent provides visual feedback if the patient exceeds their previous record. Once the patient completes the exercise, the result is provided, and they can listen to their recorded voice and decide to repeat the exercise. |
| 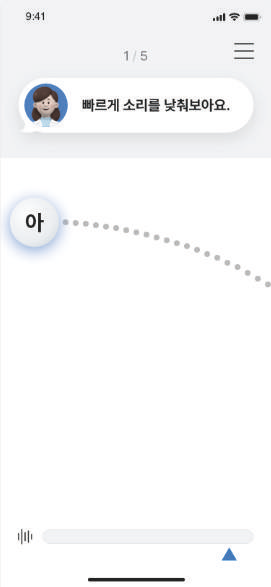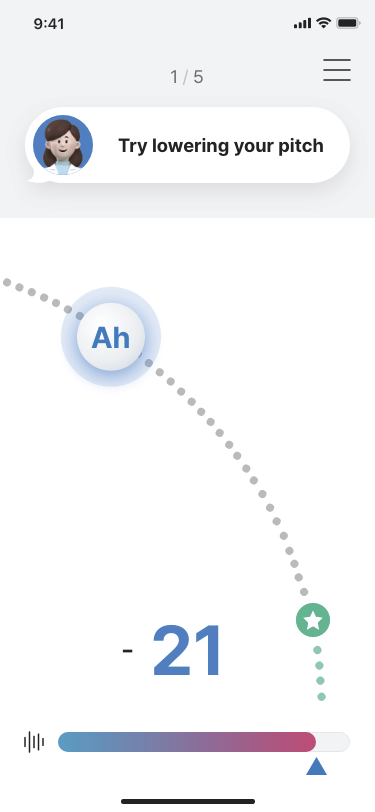 | **5. Gliding down**  This exercise requires the patient to quickly glide down the target sound (e.g., /ah/). During the training, real-time feedback of frequency (Hz) is provided. The agent provides visual feedback if the patient exceeds their previous record. Once the patient completes the exercise, the result is provided, and they can listen to their recorded voice and decide to repeat the exercise. |
| 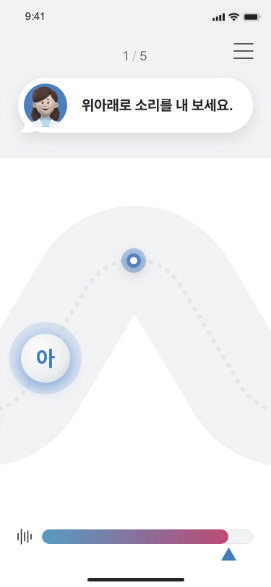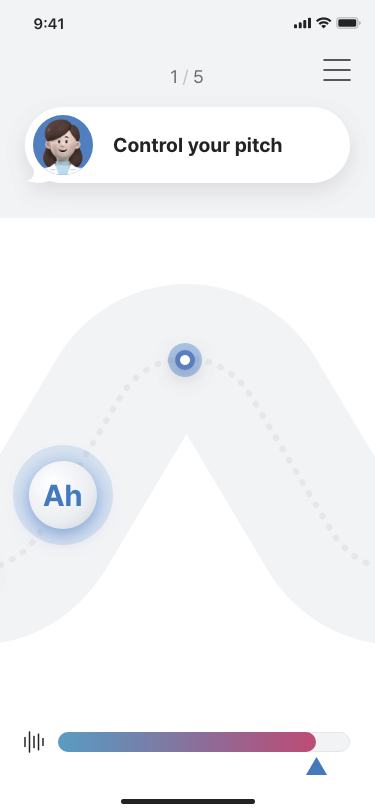 | **6. Pitch control**  This exercise requires the patient to repeatedly adjust the pitch of the target sound (e.g., /ah/). The agent provides visual feedback on success or failure each time the pitch is appropriately adjusted. Once the patient completes the exercise, the result is provided, and they can listen to their recorded voice and decide to repeat the exercise. |
| 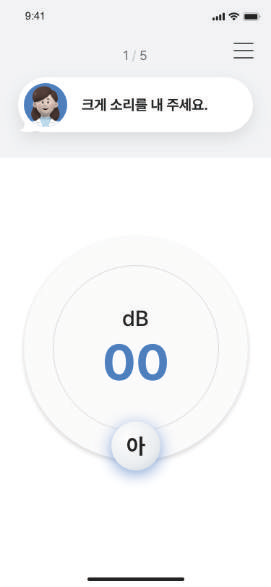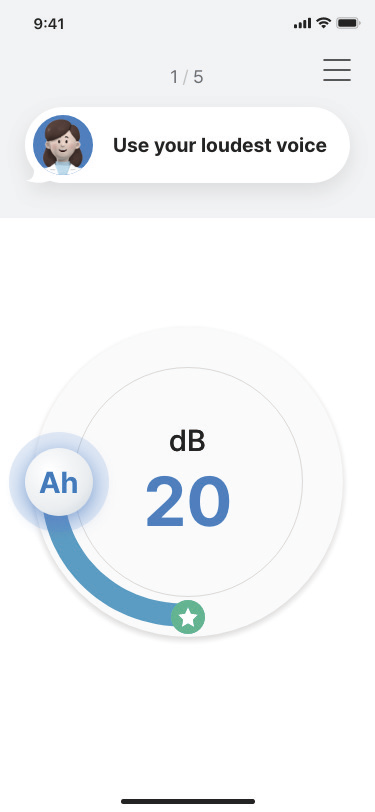 | 7. Volume up  This exercise requires the patient to loudly say the target sound (e.g., /ah/). During the training, real-time feedback is provided. Once the patient reaches the target loudness, visual feedback is given, and the training automatically ends. |
| 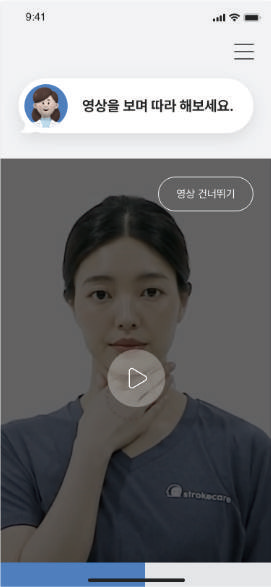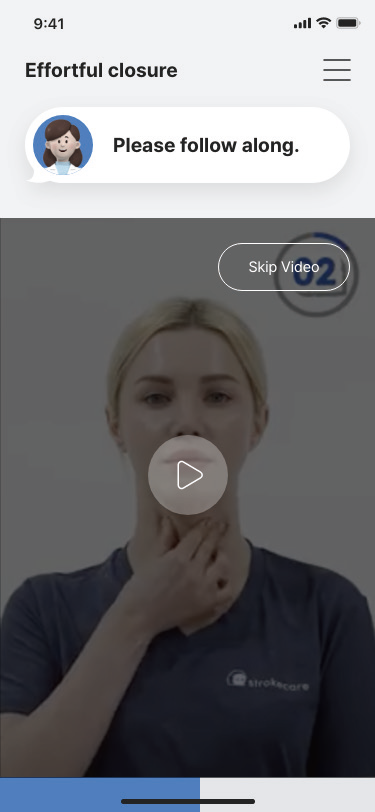 | **8. Effortful closure exercises**  This exercise provides instructional videos for an effortful closure. The patient should be able to pause, skip, and play each video. Once the patient watches all the videos, he/she can watch the selected videos again. |
| 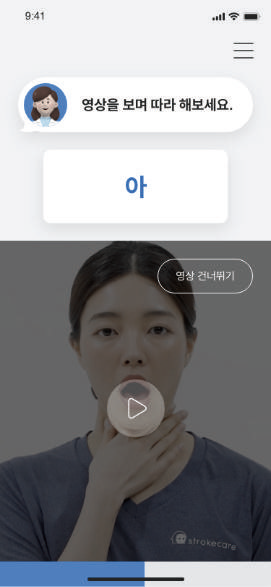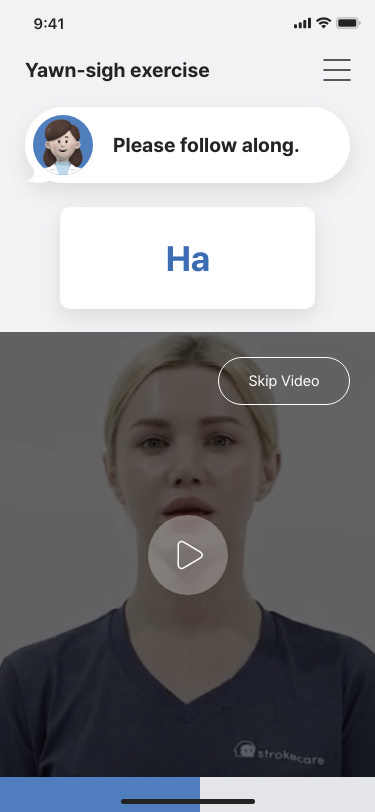 | **9. Yawn–sigh exercises**  This exercise provides instructional videos for yawn–sigh. The patient should be able to pause, skip, and play each video. Once the patient watches all the videos, he/she can watch the selected videos again. |
| 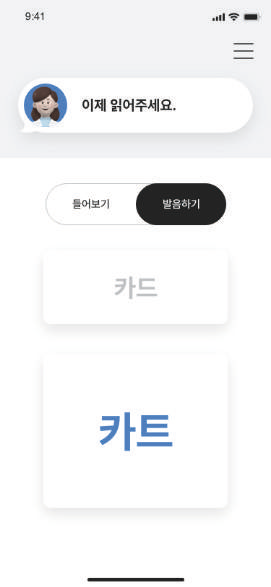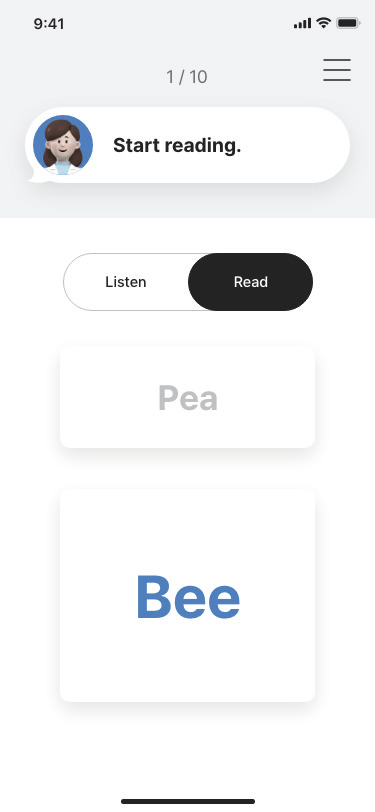 | **10. Contrast drills**  This exercise requires the patient to listen to the agent’s demonstration and read the paired words three times. Once the patient completes the exercise, they can listen to their recorded voice and decide to repeat the exercise. |
| 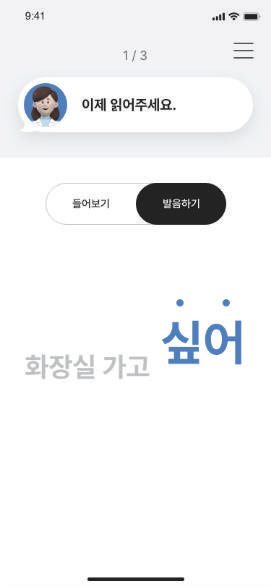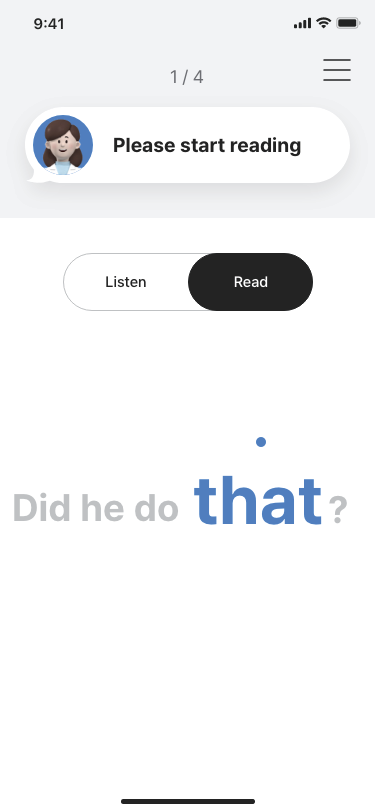 | **11. Contrastive stress**  This exercise requires the patient to listen to the agent’s demonstration and read the provided sentences. Visual feedback is provided for words in the sentence requiring stress. Once the patient completes the exercise, they can listen to their recorded voice and decide to repeat the exercise. |
| 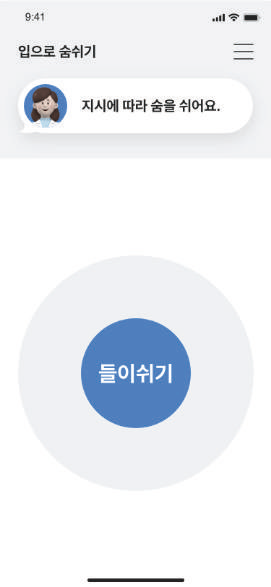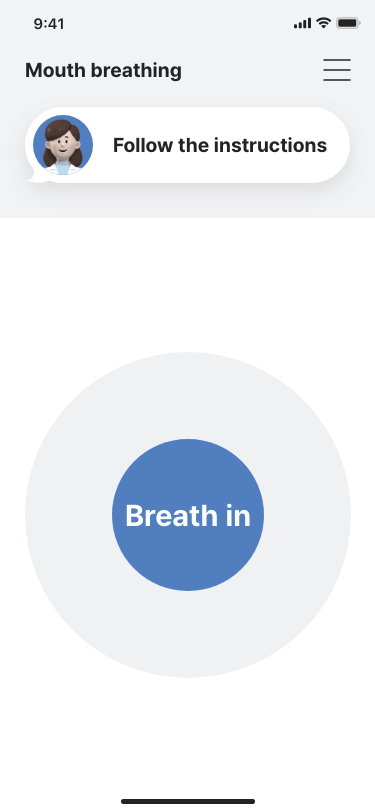 | **12. Breathing exercises**  This exercise requires the patient to practice breathing through the mouth and nose separately. According to the agent’s guidance, the patient can deeply inhale and exhale for a given amount of time. Once the patient completes the exercise, they can decide to repeat the exercise. |
| 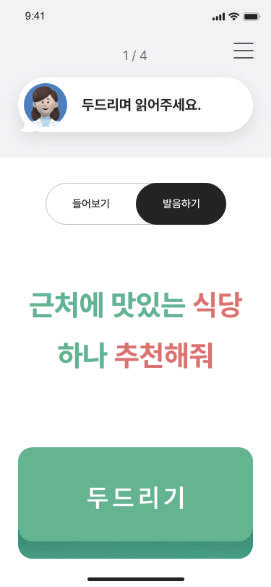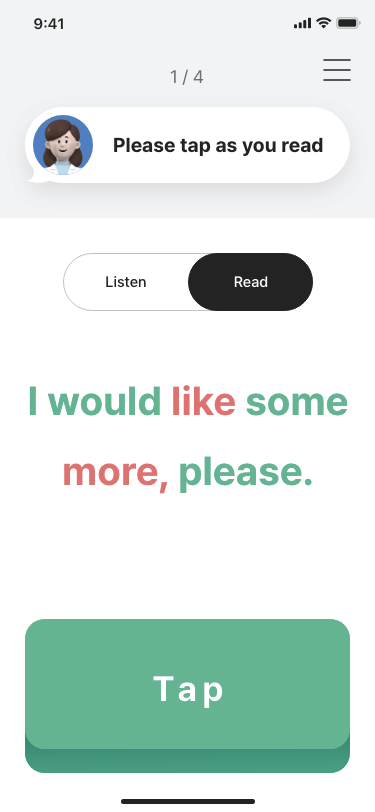 | **13. Tapping**  In this exercise, patients are prompted to produce sounds in sync with a predetermined rhythm. To aid in maintaining the rhythm, they receive visual cues about when to tap and read. Once the patient completes the exercise, their performance results will be provided, and they can listen to their recorded voice for self-evaluation. |
| 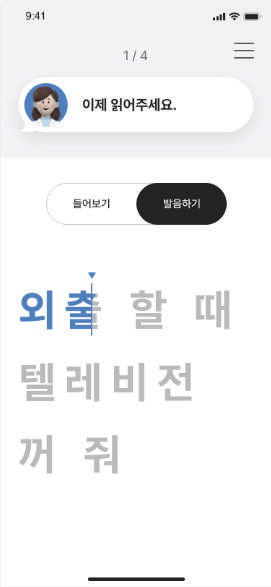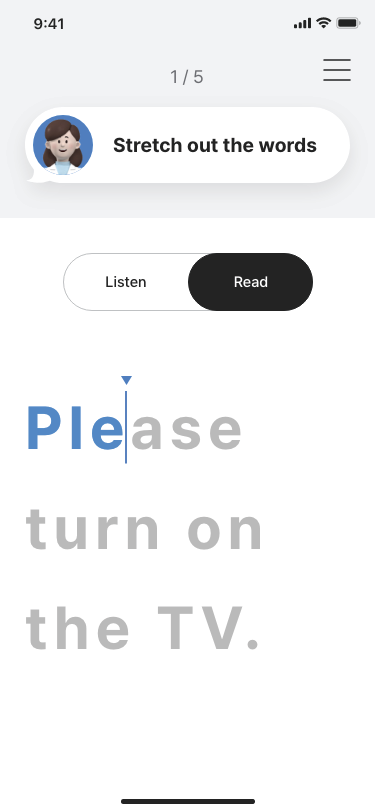 | **14. Slow reading**  This exercise requires the patient to listen to the agent’s demonstration and read the provided sentences. The patient is provided with visual feedback to help them maintain the targeted speed. Once the patient completes the exercise, they can listen to their recorded voice and decide to repeat the exercise. |
| 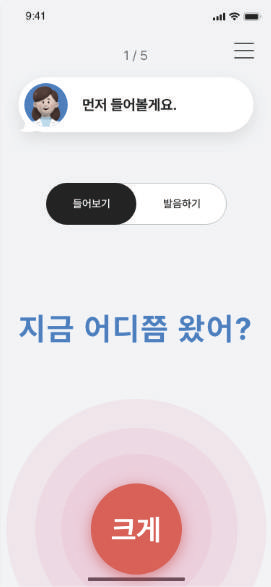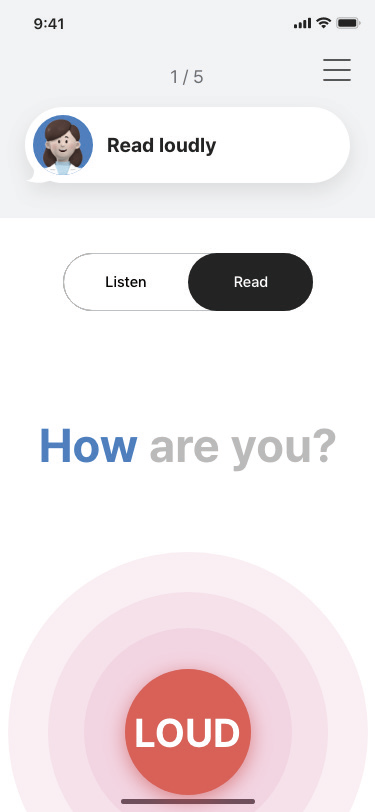 | **15. Loud reading**  This exercise requires the patient to listen to read the provided sentences at a normal volume and then read the same sentences as loudly as possible. Once the patient completes the exercise, they can listen to their recorded voice and decide to repeat the exercise. |
